# The 5-HT_1B_ receptor - a potential target for antidepressant treatment

**DOI:** 10.1007/s00213-018-4872-1

**Published:** 2018-03-15

**Authors:** Mikael Tiger, Katarina Varnäs, Yoshiro Okubo, Johan Lundberg

**Affiliations:** 1 Department of Clinical Neuroscience, Center for Psychiatry Research, Karolinska Institutet and Stockholm County Council, Centrum för psykiatriforskning, R5:0, Karolinska Universitetssjukhuset i Solna, -171 76 Stockholm, SE Sweden; 2 Department of Neuropsychiatry, Graduate School of Medicine, Nippon Medical School, Tokyo, Japan

**Keywords:** 5-HT_1B_ receptors, Serotonin, Depression, Antidepressants

## Abstract

Major depressive disorder (MDD) is the leading cause of disability worldwide. The serotonin hypothesis may be the model of MDD pathophysiology with the most support. The majority of antidepressants enhance synaptic serotonin levels quickly, while it usually takes weeks to discern MDD treatment effect. It has been hypothesized that the time lag between serotonin increase and reduction of MDD symptoms is due to downregulation of inhibitory receptors such as the serotonin 1B receptor (5-HT1BR). The research on 5-HT1BR has previously been hampered by a lack of selective ligands for the receptor. The last extensive review of 5-HT1BR in the pathophysiology of depression was published 2009, and based mainly on findings from animal studies. Since then, selective radioligands for in vivo quantification of brain 5-HT1BR binding with positron emission tomography has been developed, providing new knowledge on the role of 5-HT1BR in MDD and its treatment. The main focus of this review is the role of 5-HT1BR in relation to MDD and its treatment, although studies of 5-HT1BR in obsessive-compulsive disorder, alcohol dependence, and cocaine dependence are also reviewed. The evidence outlined range from animal models of disease, effects of 5-HT1B receptor agonists and antagonists, case-control studies of 5-HT1B receptor binding postmortem and in vivo, with positron emission tomography, to clinical studies of 5-HT1B receptor effects of established treatments for MDD. Low 5-HT1BR binding in limbic regions has been found in MDD patients. When 5-HT1BR ligands are administered to animals, 5-HT1BR agonists most consistently display antidepressant-like properties, though it is not yet clear how 5-HT1BR is best approached for optimal MDD treatment.

## Introduction

Major depression is a significant contributor to the global burden of disease, and likely the leading cause of disability in the industrialized world (Whiteford et al. 2013). Although major depressive disorder (MDD) is a highly treatable condition, half of the patients fail to respond to treatment with a selective serotonin reuptake inhibitor (SSRI), the first line of pharmacological treatment for MDD. Furthermore, with most antidepressants there is a lag time of weeks between initiation of treatment and significant antidepressant effect (Gelenberg and Chesen 2000). A majority of drugs for depression target the serotonin system with increased serotonin concentrations as a common effect, which has been the main rationale for the serotonin hypothesis of depression, stating that depression may be due to serotonin deficiency in the brain (Lapin and Oxenkrug 1969). In the absence of noninvasive methods to directly assess brain serotonin levels, in vivo confirmatory support for this hypothesis is lacking. However, recent molecular imaging techniques allowing for the study of brain neurotransmitter receptors in vivo have provided new knowledge regarding the involvement of receptors for serotonin in the pathophysiology and treatment of MDD.

Since the serotonin-enhancing effect of antidepressants such as SSRI has a rapid onset (Rothman and Baumann 2002), it has been suggested that subsequent downstream receptor regulation might be important for the antidepressant effect (Nutt 2002). Out of the 14 receptors for serotonin (5-hydroxytryptamine, 5-HT), the inhibitory 5-hydroxytryptamine_1A_ (5-HT_1A_) and 5-hydroxytryptamine_1B_ (5-HT_1B_) receptors have attracted particular attention as potentially involved in the pathophysiology of depression and as putative targets in the pharmacologic treatment of MDD (Moret and Briley 2000; Murrough et al. 2011b; Murrough and Neumeister 2011; Ruf and Bhagwagar 2009; Sari 2004; Savitz et al. 2009; Tiger et al. 2014).

5-HT_1A_ and 5-HT_1B_ receptors display 43% amino acid sequence homology and belong to the same family of G protein-coupled receptors (Hoyer et al. 2002). Importantly, the receptor subtypes display distinct cellular localizations, with 5-HT_1A_ receptors being confined to somata and dendrites (Sotelo et al. 1990), and 5-HT_1B_ receptors localized predominantly in axon terminals (Boschert et al. 1994). Activation of 5-HT_1A_ and 5-HT_1B_ receptors in serotonergic neurons thereby serves to regulate extracellular 5-HT levels by different mechanisms, i.e., by controlling neuronal firing rate (Sprouse and Aghajanian 1987) and by modulating transmitter release (Engel et al. 1986; Middlemiss 1984), respectively. In addition, notable differences in the distribution of postsynaptic 5-HT_1A_ and 5-HT_1B_ receptors in nonserotonergic neurons further support distinct functional roles of these receptors. While 5-HT_1A_ receptors are abundantly localized in cortical regions (Hall et al. 1997), 5-HT_1B_ receptors are widely distributed in the brain, showing particularly high density in the basal ganglia (see “5-HT_1B_ receptor brain distribution” section).

The role of 5-HT_1A_ receptors in the pathophysiology and treatment of depression has been thoroughly investigated (Savitz et al. 2009). However, research on 5-HT_1B_ receptors has earlier been hampered by a lack of selective ligands (Barnes and Sharp 1999; Middlemiss and Hutson 1990). Recent development of compounds with 5-HT_1B_ receptor selectivity has paved the way for exploration of the role of 5-HT_1B_ receptors in MDD (Slassi 2002; Zimmer and Le Bars 2013).

This is a review of the relevance of 5-HT_1B_ receptors in psychiatric disorders, especially in MDD. Thorough literature searches applying MeSH terms have been conducted in PubMed, on “5-HT_1B_ receptor AND depression,” “5-HT_1B_ receptor AND anxiety,” “5-HT_1B_ receptor AND aggression,” “5-HT_1B_ receptor AND alcohol,” and “5-HT_1B_ receptor AND cocaine,” for an overview of the field.

## 5-HT_1B_ receptor structure and intracellular function

The 5-HT_1B_ receptor has a putative seven transmembrane spanning structure (Saudou and Hen 1994) and is G_i_-protein coupled (Barnes and Sharp 1999; Hamblin et al. 1987), inhibiting adenylate cyclase as demonstrated with reduced forskolin-stimulated cAMP release upon agonist binding (Adham et al. 1992; Levy et al. 1992a; Weinshank et al. 1992). The gene coding for the mouse 5-HT_1B_ receptor is located on chromosome 9 (position 9E), and the human 5-HT_1B_ receptor gene is located on chromosome 6 (6q13) (Saudou and Hen 1994). The amino acid sequence of the 5-HT_1B_ receptor gene is to a high degree similar for humans and rodents, with 93% overall homology and 96% homology in transmembrane regions (Adham et al. 1992; Maroteaux et al. 1992; Voigt et al. 1991). Despite this genetic similarity, the species variants of 5-HT_1B_ receptors display some distinct differences in drug affinity, with higher affinity for the selective 5-HT_1B/1D_ receptor agonist sumatriptan and lower affinity for β-adrenergic receptor antagonists such as pindolol and propranolol for the human 5-HT_1B_ receptor compared to rodent variants (Adham et al. 1992; Demchyshyn et al. 1992; Hamblin et al. 1992; Jin et al. 1992; Levy et al. 1992b; Weinshank et al. 1992; Voigt et al. 1991). This species difference seems mainly conferred by a single amino acid. Replacement of threonine at residue 355, on the seventh transmembrane segment, with the corresponding asparagine in rodents renders the human 5-HT_1B_ receptor essentially the same pharmacological properties as the rodent receptor (Metcalf et al. 1992; Oksenberg et al. 1992; Parker et al. 1993).

### 5-HT_1B_ receptor distribution and function

The 5-HT_1B_ receptor is involved in a broad repertoire of physiological effects, including satiety (Voigt and Fink 2015), sleep (Boutrel et al. 1999), locomotor activity (Chaouloff et al. 1999; Cheetham and Heal 1993; Ramboz et al. 1996), sexual behavior and ejaculatory function (Giuliano 2007; Rodriguez-Manzo et al. 2002), reduction of body temperature (Hagan et al. 1997), and modulation of memory and learning (Buhot et al. 2000). The main body of the 5-HT_1B_ receptor literature relates to its potential role in psychiatric disorders, which will be outlined in more detail in “The involvement of 5-HT_1B_ receptors in behaviors relevant to psychiatry” section.

The cellular localization of 5-HT_1B_ receptors is mainly presynaptic, with receptors distributed primarily to axon terminals, as demonstrated with autoradiography, lesion studies, immunocytochemistry, and viral transfection studies (Boschert et al. 1994; Bruinvels et al. 1994; Sari 2004; Varnas et al. 2005). Depending on localization, 5-HT_1B_ receptors may act as autoreceptors, inhibiting serotonin release (Barnes and Sharp 1999; Brazell et al. 1985; Buhlen et al. 1996; Davidson and Stamford 1995; De Groote et al. 2003; Engel et al. 1986; Hjorth and Tao 1991; Limberger et al. 1991; Martin et al. 1992; Middlemiss 1984; Rutz et al. 2006; Schlicker et al. 1997; Sharp et al. 1989; Starkey and Skingle 1994), or as heteroreceptors, regulating the release of other transmitters (Barnes and Sharp 1999; Ruf and Bhagwagar 2009; Sari 2004).

#### 5-HT_1B_ autoreceptors

In the serotonin projection areas, the role of 5-HT_1B_ receptors in regulation of 5-HT release is relatively straightforward, and inhibitory. Upon binding to the 5-HT_1B_ receptors, 5-HT inhibits formation of cAMP and downstream cellular responses. This results in diminished transmitter release (Barnes and Sharp 1999; Leenders and Sheng 2005; Middlemiss and Hutson 1990). More recent research supports that the 5-HT_1B_ receptors can regulate serotonin transporter function, thus serving as an additional mechanism by which 5-HT_1B_ autoreceptors modulate extracellular transmitter levels in serotonergic projection regions (Hagan et al. 2012; Montanez et al. 2014). Administration of the 5-HT_1B_ receptor agonist CP-93,129 suppressed 5-HT release in the hippocampus in rats (Hjorth and Tao 1991). In wild-type mice, 5-HT_1B_ receptor agonist-induced decrease and 5-HT_1B_ receptor antagonist-induced increase of 5-HT in the hippocampus and cortex has been demonstrated, whereas no effects were found in 5-HT_1B_ receptor gene knockout mice (Rutz et al. 2006).

In the raphe nuclei, the regulation of the serotonin system by 5-HT_1B_ receptors is more complex (Sari 2004). Local perfusion with the 5-HT_1B_ receptor agonist CP-93,129 decreased 5-HT release in the dorsal and median raphe nucleus in rats (Adell et al. 2001). In the same study with local perfusion of another 5-HT_1B_ receptor agonist, CP-94,253, a biphasic effect on 5-HT release was found in the median raphe nucleus, with 5-HT reductions at low doses and increase of 5-HT at a high dose of CP-94,253 (Adell et al. 2001). The biphasic regulation of 5-HT release may suggest the presence of 5-HT_1B_ receptors both in serotonin neurons and inhibitory neurons controlling the release of 5-HT in the median raphe nuclei (Adell et al. 2001; Bagdy et al. 2000).

Recent studies in mouse models developed for tissue-specific regulation of 5-HT_1B_ expression have provided further insight regarding the functional role of 5-HT_1B_ autoreceptors. Selective knockdown of the 5-HT_1B_ autoreceptors was found to increase extracellular 5-HT levels in response to an SSRI and to induce antidepressant-like phenotypes, thus supporting the potential benefit of pharmacologic inhibition of these receptors for treatment of depression (Nautiyal et al. 2016). The role of 5-HT_1B_ receptor antagonists in the treatment of depression will be further described in the “Effects of agonists and antagonists in animal models of depression” section.

#### 5-HT_1B_ heteroreceptors

With 5-HT_1B_ receptors located on nonserotonergic neurons regulation of the release of glutamate, GABA, acetylcholine, and dopamine has been demonstrated (Ruf and Bhagwagar 2009). There is some, but relatively sparse, data on the effect of 5-HT_1B_ receptors on glutamate, the major excitatory transmission system in the brain. A subpopulation of 5-HT_1B_ receptors were shown to be colocalized with the AMPA receptor subunit GluR2 in hippocampal dentate gyrus in rats, as visualized with immunofluorescence in dendrites (Peddie et al. 2010). Recently, our group reported a correlation between 5-HT_1B_ receptor binding and glutamatergic N-methyl-d-aspartate receptor binding in layers I–III of the anterior cingulate cortex (ACC) of human postmortem tissue (Veldman et al. 2017). Functionally, there are indirect signs of decreased glutamate release by presynaptic 5-HT_1B_ receptors, with selectively reduced amplitude of evoked excitatory postsynaptic currents in the bed nucleus of the stria terminalis (BNST) by the 5-HT_1B_ the receptor agonist CP93,129 (Guo and Rainnie 2010).

There are more data supporting a role of 5-HT_1B_ receptors in regulating inhibitory transmitter systems. A number of studies demonstrate an inhibitory effect of the 5-HT_1B_ receptor on the main inhibitor in the brain, gamma-aminobutyric acid (GABA). The 5-HT_1B/1D_ receptor agonist sumatriptan inhibited GABA release in the neocortex (Feuerstein et al. 1996). Inhibition of GABA-induced inhibitory postsynaptic currents (ISPCs) by 5-HT_1B_ receptors has been demonstrated with patch clamp recordings in substantia nigra, suprachiasmatic nuclei, subthalamic nucleus, and globus pallidus (Bramley et al. 2005; Chen et al. 2008; Ding et al. 2015; Hashimoto and Kita 2008; Shen and Johnson 2008; Stanford and Lacey 1996). There is also immunohistochemical evidence of 5-HT_1B_ acting as a heteroreceptor on the GABA system, with a majority of GABA-positive neurons in the inferior colliculus also being positive for the 5-HT_1B_ receptor (Peruzzi and Dut 2004).

Furthermore, 5-HT_1B_ receptors on cholinergic neurons inhibit acetylcholine release in the ventral striatum (Virk et al. 2016) and hippocampus (Cassel et al. 1995; Maura and Raiteri 1986). In a microdialysis study, administration of the 5-HT_1B_ receptor antagonist NAS-181 increased acetylcholine levels profoundly in the frontal cortex and hippocampus, and based on this finding the authors suggested that acetylcholine release in these brain regions is under tonic inhibitory control by 5-HT_1B_ receptors (Hu et al. 2007).

Dopaminergic neurons are also regulated by 5-HT_1B_ receptors, with mostly increased dopamine release upon receptor activation in nigrostriatal, mesolimbic, and mesocortical pathways, as previously reviewed (Alex and Pehek 2007). Given the G_i_ protein-coupled inhibitory function of 5HT_1B_ receptors and the absence of 5-HT_1B_ receptor mRNA expression on dopaminergic neurons in the substantia nigra, and ventral tegmentum (Bruinvels et al. 1994; Sari et al. 1999; Varnas et al. 2005), it is not likely that the facilitative effect on dopamine transmission is mediated by 5-HT_1B_ receptors residing on dopaminergic neurons. More plausible instead is indirect 5-HT_1B_ receptor action on dopaminergic neurons, via inhibition of dopamine restricting GABAergic interneurons in ventral tegmentum (O'Dell and Parsons 2004; Yan et al. 2004), yielding increased dopamine concentrations. Indeed, 5-HT_1B_ receptor agonists have been reported to reduce the GABA_B_ synaptic potential in dopamine neurons in rats (Johnson et al. 1992).

### 5-HT_1B_ receptor brain distribution

**Fig. 1:**
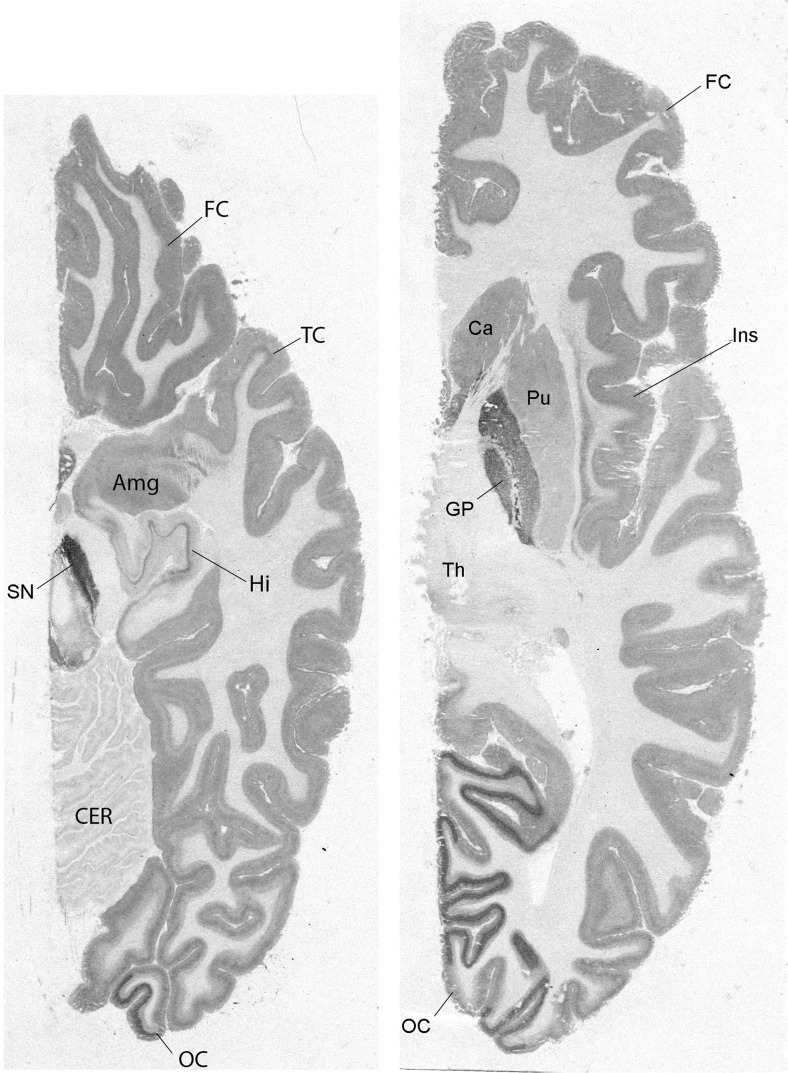
Autoradigraphic mapping of 5-HT_1B_ receptors in the human brain. Images show binding of the radioligand [^3^H]GR125743 in brain sections at the level of substantia nigra (left) and globus pallidus (right). Amg, amygdala; Ca, caudate nucleus; CER, cerebellum; FC, frontal cortex; GP, globus pallidus; Hi, hippocampus; Ins, insular cortex; OC, occipital cortex; Pu, putamen; SN, substantia nigra; TC, temporal cortex; Th, thalamus

**Fig. 2:**
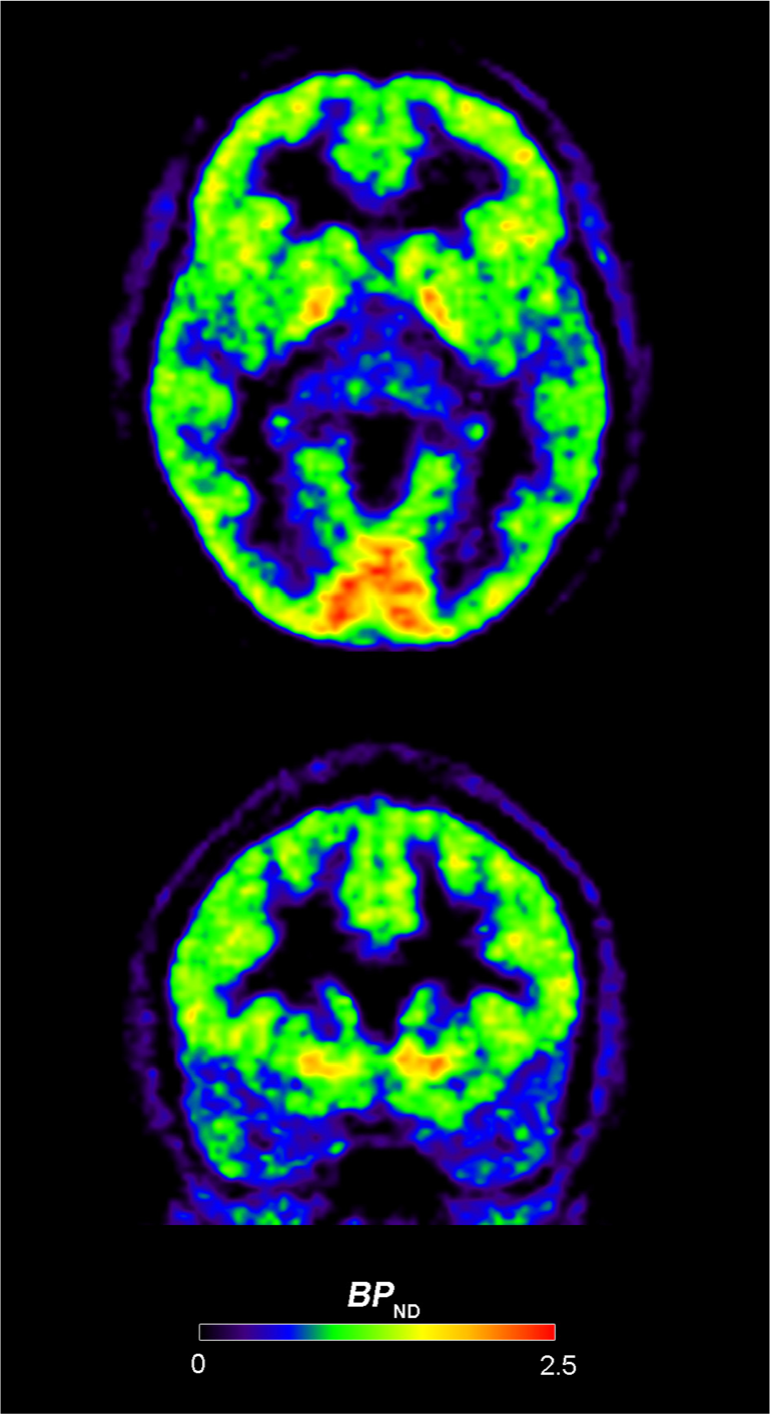
PET images of the distribution of 5-HT_1B_ receptors in human brain. Average images of binding potential (*BP*_ND_) for the radioligand [^11^C]AZ10419369 in eight control subjects

The distribution of 5-HT_1B_ receptors in the brain has been mapped with autoradiography (Fig. 1). In humans, high 5-HT_1B_ receptor densities have been found in the substantia nigra and globus pallidus (Bonaventure et al. 1997; Varnas et al. 2001). Intermediate densities of 5-HT_1B_ receptors were found in the striatum, with higher binding in ventromedial parts, the dorsal raphe nucleus, and the cerebral cortex, although there was a subregion within the medial occipital cortex with denser labeling (Varnas et al. 2001; Varnas et al. 2004). Lower 5-HT_1B_ receptor binding was found in the hippocampus and the amygdala (Varnas et al. 2001). Low 5-HT_1B_ receptor density was observed in the thalamus and very low (Varnas et al. 2001) or no (Bonaventure et al. 1997). 5-HT_1B_ receptor binding was found in the cerebellum. This rank order of 5-HT_1B_ receptor brain distribution has been confirmed in vivo with positron emission tomography (PET) (Fig. 2) and the 5-HT_1B_ receptor selective radioligand [^11^C]AZ10419369 (Varnas et al. 2011). It is homologous with the distribution in rodents, with high 5-HT_1B_ receptor levels in substantia nigra and globus pallidus (Bruinvels et al. 1993; Langlois et al. 1995) and intermediate levels in the striatum (Bruinvels et al. 1993).

Regional 5-HT_1B_ receptor protein synthesis in the brain has been mapped with in situ hybridization histochemistry, with antisense-RNA probes to determine regional mRNA expression. In most examined regions in humans, mRNA expression levels matched the reported distribution of 5-HT_1B_ receptors, with relatively high expression in the caudate nucleus, putamen, ventral striatum, cerebral cortex, and rostral raphe nuclei (Bidmon et al. 2001; Jin et al. 1992; Varnas et al. 2005). However, there was a distinct mismatch in the globus pallidus and substantia nigra, 5-HT_1B_ receptor-rich regions where no 5-HT_1B_ receptor mRNA expression was found. Absence of 5-HT_1B_ receptor mRNA expression in the globus pallidus and substantia nigra despite high 5-HT_1B_ receptor densities in these regions appears across species, reported in rodents as well as guinea pigs (Bonaventure et al. 1998; Boschert et al. 1994). This mismatch, with high 5-HT_1B_ receptor levels not accompanied by corresponding regional protein synthesis, is one in a line of evidences of axon terminal localization of the 5-HT_1B_ receptor (Sari 2004; Varnas et al. 2005).

When interpreting imaging findings based on studies with 5-HT_1B_ receptor radioligands, it is important to note that the radioligands do not distinguish between the functionally different autoreceptors and heteroreceptors localized in serotonergic and nonserotonergic neurons. However, given the abundant 5-HT_1B_ mRNA expression in forebrain regions having high receptor density, it is likely that the heteroreceptor population represents a notable proportion of forebrain 5-HT_1B_ receptors. A major contribution of the heteroreceptors to the signal of 5-HT_1B_ receptor radioligands is further supported by recent findings of markedly reduced binding after selective knockdown of 5-HT_1B_ heteroreceptors in mice (Nautiyal et al. 2015). By contrast, neurotoxic lesioning of serotonergic neurons (Compan et al. 1998) or selective ablation of 5-HT_1B_ autoreceptors (Nautiyal et al. 2015) has no detectable effect on radioligand binding, suggesting that the autoreceptor component represents a minor proportion of forebrain 5-HT_1B_ receptors.

### The relationship of the 5-HT_1B_ receptor to p11

5-HT_1B_ receptor function and distribution is intimately linked to the intracellular protein p11 (S100A10) (Svenningsson et al. 2006). p11 is required for translocation to the plasma membrane of a number of intracellular proteins, such as Annexin II (Deora et al. 2004), the sodium channel Na_V_1.8/SNS (Okuse et al. 2002), the calcium channels TRPV5 and TRPV6 (van de Graaf et al. 2003), and the potassium channel TASK-1 (Girard et al. 2002). Trafficking of 5-HT_1B_ receptors to the plasma membrane is also dependent on p11, with twofold higher surface receptor portions in COS-7 cells cotransfected with p11 compared to cells only transfected with 5-HT_1B_ receptors (Svenningsson et al. 2006). Moreover, the inhibitory effect of serotonin on forskolin-induced cAMP formation was significantly enhanced in p11 cotransfected cells (Svenningsson et al. 2006). In line with this, absence of p11, as demonstrated in a p11 gene knockout mice model, resulted in low 5-HT_1B_ receptor binding in the globus pallidus and substantia nigra (Svenningsson et al. 2006). In the p11 knockout mice, 5-HT_1B_ receptor agonist-induced cortical downregulation of phosphor-Thr^202^Tyr^204^-ERK1/2 was lost and inhibition of cAMP formation upon 5-HT_1B_ receptor agonist administration was reduced, as demonstrated with phosphor-Ser^9^-synapsin I levels in striatum (Svenningsson et al. 2006). Furthermore, interaction between p11 and 5-HT_1B_ receptors has been confirmed with Western blotting, where coimmunoprecipitation was found in HeLa cells and mouse brain tissue (Svenningsson et al. 2006). Colocalization of p11 and 5-HT_1B_ receptors at the cell surface has been demonstrated with immunofluorescence staining (Svenningsson et al. 2006). Moreover, in mouse brain distribution of mRNA expression is remarkably similar for p11 and 5-HT_1B_ receptors (Svenningsson et al. 2006). This impression has been confirmed with immunohistochemically labeled cells, where p11 and 5-HT_1B_ receptors were coexpressed in the striatum, hippocampus, and cingulate cortex (Egeland et al. 2011). In humans, significant correlations between p11 and 5-HT_1B_ receptor mRNA levels in the orbitofrontal cortex, frontopolar cortex, and hippocampus postmortem have been reported in suicide subjects as well as controls (Anisman et al. 2008). Thus, p11 plays an important role in 5-HT_1B_ receptor expression and function; although this interaction is not specific, p11 also modulates surface expression and function of the 5-HT_4_ receptor (Warner-Schmidt et al. 2009) and mGluR5 (metabotropic glutamate receptor 5 (Lee et al. 2015)).

## The involvement of 5-HT_1B_ receptors in behaviors relevant to psychiatry

### 5-HT_1B_ receptors in relation to anxiety

Knockout of the 5-HT_1B_ receptor gene results in mice with a distinct behavioral phenotype, aggressive and less cautious (Saudou et al. 1994; Zhuang et al. 1999). Decreased anxiety in the 5-HT_1B_ receptor knockout mice has consequently been reported, with low anxiety in the elevated plus maze and more activity in the open field (Brunner et al. 1999; Nautiyal et al. 2016; Zhuang et al. 1999). In line with this, overexpression of 5-HT_1B_ receptors in the dorsal raphe nucleus has yielded increased stress-induced anxiety in rats (Clark et al. 2002). In contrast, the 5-HT_1B_ receptor-overexpressing rats displayed less anxiety, before exposure to stress (Clark et al. 2002). Furthermore, in nonstressed rats, there was an inverse correlation between 5-HT_1B_ receptor mRNA expression in dorsal raphe nucleus and anxiety, as estimated with activity in the elevated plus maze (Kaiyala et al. 2003). In serotonergic projection areas in the brain, high 5-HT_1B_ receptor density has been reported in a genetic mouse model for anxiety (Clement et al. 1996).

Another major source of information about the behavioral effects of 5-HT_1B_ receptors stems from extrapolation of the effects of 5-HT_1B_ receptor ligands. Most reports describe anxiogenic effects of 5-HT_1B_ receptor agonists (Sari 2004), with increased anxiety in paradigms as the elevated plus-maze (Lin and Parsons 2002; Solati et al. 2011). Sari hypothesized that 5-HT_1B_ receptors modulate anxiety through inhibition of serotonin, acetylcholine, and GABA (Sari 2004). However, anxiolytic properties of 5-HT_1B_ receptor agonists have also been reported. The early reports on reduced anxiety upon 5-HT_1B_ receptor activation have been dismissed as results of nonselective ligands (Sari 2004). Still, reduced anxiety-related behavior has been demonstrated with CP-94,253, a selective 5-HT_1B_ receptor agonist, in the Vogel conflict drinking test, which is sensitive to the acute anxiolytic effects of bensodiazepines, but fail to detect SSRI effect (Chojnacka-Wojcik et al. 2005). Furthermore, CP-94,253 attenuated the anxiogenic effects of cocaine (Klein et al. 2016), while the 5-HT_1B_ receptor antagonist increased cocaine-induced anxiety (Hoplight et al. 2005). Otherwise, in baseline conditions, the effect of 5-HT_1B_ receptor antagonists is more clear and consequent. Anxiolytic properties of 5-HT_1B_ receptor antagonists has been demonstrated in a variety of paradigms for anxiety such as Vogel drinking test (Chojnacka-Wojcik et al. 2005), separation-induced vocalization (Dawson et al. 2006; Hudzik et al. 2003; Zhang et al. 2011), human threat test (Dawson et al. 2006), and reactions to a novel environment (Nowicki et al. 2014). In humans, studies on the role of 5-HT_1B_ receptors in anxiety are disproportionately sparse. Increased fear of public speaking has been reported in healthy volunteers after administration of the 5-HT_1B/1D_ receptor agonist sumatriptan (de Rezende et al. 2013). In the single in vivo brain imaging study of subjects with posttraumatic stress disorder, low 5-HT_1B_ receptor binding was described in the caudate, amygdala, and anterior cingulate cortex (ACC). The significant differences in binding compared with control were driven by the low 5-HT_1B_ receptor binding potential (*BP*_ND_) in a subgroup of subjects with major depressive disorder comorbidity (Murrough et al. 2011a).

Another condition in which 5-HT_1B_ receptors have been suggested to be involved in the etiology and pathophysiology is obsessive-compulsive disorder (OCD), which has a complex, though fundamental, relation to anxiety. In genetic studies on 5-HT_1B_ receptors in OCD, an association between the G861C polymorphism in the 5-HT_1B_ receptor gene and OCD has been found and replicated (Kim et al. 2009; Mundo et al. 2000). However, in a study of OCD probands and parents, the silent 5-HT_1B_ receptor gene G861C polymorphism in the coding region was not transmitted to a significantly larger degree than its counterpart, in a study population with a family history of OCD for 40% of the probands (Di Bella et al. 2002). Gender differences might contribute to inconsistent results, since Kim et al. only examined men, and a broader genetic study found significant transmission of 5-HT_1B_ receptor gene polymorphisms only to male and not to female probands (Mas et al. 2014). Furthermore, a recent positron emission tomography (PET) study found no differences in cerebral 5-HT_1B_ receptor binding in OCD patients compared with controls (Pittenger et al. 2016). Still, the 5-HT_1B/1D_ receptor agonist sumatriptan has been reported to aggravate OCD symptoms in subjects with relatively low OCD symptom levels (Gross-Isseroff et al. 2004), although no effect on OCD was found in a group with more OCD symptoms, when subjects were given another 5-HT_1B/1D_ receptor agonist, zolmitriptan, with higher blood-brain barrier penetrance (Boshuisen and den Boer 2000). With a more 5-HT_1B_ receptor selective compound, RU24969, with high affinity for 5-HT_1B_ receptors and low affinity for 5-HT_1A_ receptors, and simultaneous administration of the 5-HT_1A_ receptor antagonist WAY100635, a dose-dependent increase of locomotor stereotypy has been demonstrated in a rodent model of OCD. The RU24969-exposed rats had increased *Fos* expression, an indirect marker of neuronal activity, in the dorsal striatum, a region implicated in PET studies of OCD (Saxena and Rauch 2000). This RU24969-induced increase of striatal neuronal activity was obliterated with SSRI pretreatment (Ho et al. 2016). Altogether, animal data imply a role for the 5-HT_1B_ receptor in anxiety, though human studies are sparse and inconclusive.

### 5-HT_1B_ receptors in relation to depressive states

Depression is the psychiatric condition with most reports in the literature in relation to the 5-HT_1B_ receptor. A large part of previous 5-HT_1B_ receptor depression research stems from preclinical studies, mostly in rodents (Ruf and Bhagwagar 2009). Mice constitutionally or conditionally genetically deprived of 5-HT_1B_ receptors not only are less anxious, but also show less depression-like behavior, with less immobility time in both the forced swim test (FST) and the tail suspension test (TST) (Jones and Lucki 2005; Nautiyal et al. 2016), and higher sucrose preference (Bechtholt et al. 2008; Nautiyal et al. 2016), Jones and Lucki found significantly lower immobility time only in female 5-HT_1B_ receptor knockout mice compared to wild-type mice. Furthermore, in a number of microdialysis studies, an augmentation of serotonin levels in response to SSRI was found in the hippocampus (Knobelman et al. 2001; Malagie et al. 2001; Nautiyal et al. 2016), but not in the striatum (De Groote et al. 2003; Knobelman et al. 2001) of 5-HT_1B_ receptor knockout mice compared with controls. The regional difference in SSRI-induced serotonin release may be due to innervation, with hippocampus receiving serotonin input mainly from the 5-HT_1B_ receptor key region the median raphe nucleus, while the striatum receives projections from the dorsal raphe nucleus (Knobelman et al. 2001; Tork 1990). By contrast, the knockout for the 5-HT_1B_ receptor-related p11 gene has resulted in a depressive phenotype, with more immobility time and lower preference to sucrose than wild-type littermates (Svenningsson et al. 2006). On the other hand, 5-HT_1B_ receptor binding in the p11 knockout mice is reduced, but not depleted (Svenningsson et al. 2006). This more moderate reduction in 5-HT_1B_ receptor levels would be in line with human case-control studies, in which globally low brain binding and mRNA expression has been found in patients with major depressive disorder (MDD) (Tiger et al. 2016) and suicide subjects (Anisman et al. 2008), respectively. The behavioral consequences of having low versus no 5-HT_1B_ receptors in the brain may differ considerably.

#### Animal models

The results from studies of 5-HT_1B_ receptors in animal models for depression are largely inconclusive. Low 5-HT_1B_ receptor binding has been demonstrated in the hippocampus in a rat model for inherited depressive traits, Flinder’s sensitive line, and in rats separated from their mothers. The effects of either genetic or environmental vulnerability for depression on 5-HT_1B_ receptor binding could be reversed with antidepressants (Shrestha et al. 2014). Likewise, in Rgs2-mutant mice, with long latency to eat in the novelty suppressed feeding test as the main behavioral proxy for depressed mood, raphe nuclei 5-HT_1B_ receptor gene expression was low (Lifschytz et al. 2012). On the other hand, higher 5-HT_1B_ receptor densities in most brain regions, including dorsal hippocampus and the rostral raphe nuclei, were reported in Flinder’s sensitive line rats, both compared with Flinder’s resistant line and Sprague-Dawley rats (Nishi et al. 2009). Furthermore, an early finding in the field was the twofold higher 5-HT_1B_ receptor binding in the cortex, hippocampus, and septum in rats that reacted with learned helplessness in reaction to uncontrollable electric shocks versus nonhelpless rats (Edwards et al. 1991). To complicate things further, high 5-HT_1B_ receptor mRNA in dorsal raphe nucleus has been reported both in rats with learned helplessness (Neumaier et al. 1997) as well as in stress-resilient rats (Neumaier et al. 2002).

#### Effects of agonists and antagonists in animal models of depression

The role of the 5-HT_1B_ receptor in depression-like states is more clearly disentangled in studies with drugs targeting the receptor. Blocking the inhibitory 5-HT_1B_ receptor would in theory lead to increased levels of extracellular serotonin, and potential antidepressant effects. Indeed, treatment with the 5-HT_1B/1D_ receptor antagonist GR 127935 increased latency to immobility in guinea pigs in FST (Rex et al. 2008). However, in most studies of 5-HT_1B_ receptor antagonism in depression-like states, effect has been demonstrated only with simultaneous administration of antidepressant drugs (Ruf and Bhagwagar 2009). 5-HT_1B_ receptor antagonists have been found to increase the anti-immobility effect in FST when coadministered with tricyclic antidepressants or a monoamine oxidase inhibitor (Chenu et al. 2008; Tatarczynska et al. 2004a) or with an SSRI (Tatarczynska et al. 2002). However, the 5-HT_1B/1D_ receptor antagonist GR 127935 has also been reported to block the effects of paroxetine and imipramine in mice in the tail suspension test (O'Neill et al. 1996), whereas in the same study, depression-like immobility was decreased and the antidepressant effect of imipramine was increased with the 5-HT_1B_ receptor agonist RU 24969 (O'Neill et al. 1996). Furthermore, reduced depression-like behavior has been demonstrated with different 5-HT_1B_ receptor agonists also in the forced swimming test (Chenu et al. 2008; Tatarczynska et al. 2004b). The findings that antidepressant-like effects can be obtained with both antagonists and agonists targeting the receptor likely reflect the heterogeneous localization of 5-HT_1B_ receptors in different neuronal populations where autoreceptors and heteroreceptors may differently modulate depression-like behaviors (Chenu et al. 2008; Nautiyal et al. 2016).

#### Human studies of 5-HT_1B_ receptors in MDD

Even though preclinical studies have had immense importance for the literature on 5-HT_1B_ receptors in depression, translation of animal data to the clinical diagnosis MDD poses several challenges. Although animal models of depression are sensitive to antidepressants, none of them reflect the episodic feature of MDD and the most ominous MDD symptom, suicidal ideation, is absent in other species than humans. While some animal models may mimic vulnerability to stress and thereby the characteristic disproportionate load of symptoms such as lowered mood in MDD, no model convincingly display signs of the symptoms that may persist also without precipitating external causes (Belmaker and Agam 2008). That said, it is also challenging to validly study the pathophysiology of major depressive disorder also in clinical studies, mainly due to difficulties in defining the diagnosis. The main shortcomings of the MDD definitions in the American Psychiatric Association’s Diagnostic and Statistical Manual of Mental Disorders (DSM), the most widely used MDD definition, as well as in ICD-10, is the heterogeneity of the current MDD diagnosis, which can be fulfilled in 1497 different ways (Ostergaard et al. 2011), and the disregard of current psychosocial situation. Evaluating depressive symptoms in relation to current context is most probably necessary to avoid erroneously labeling stressed subjects as depressed (Horwitz and Wakefield 2007). These flaws in the current definition of MDD may have hampered the research on pathophysiology in MDD and may explain some of the inconsistent results. Still, in the emerging picture of accumulating 5-HT_1B_ receptor MDD data, the 5-HT_1B_ serotonin receptor subtype seems relatively consistently associated with or altered in this disorder. In a study of 394 psychiatric patients, an association between the silent G861C 5-HT_1B_ receptor polymorphism, which is associated with low 5-HT_1B_ receptor brain density (Huang et al. 1999), and a history of MDD or substance abuse disorder was found (Huang et al. 2003). Moreover, there is indirect neuroendocrine evidence for altered 5-HT_1B_ receptor function in MDD, with blunted growth hormone (GH) response to the 5-HT_1B/1D_ receptor agonist sumatriptan in MDD patients (Cleare et al. 1998) and likewise reduced GH response to another triptan, zolmitriptan, which passes the blood brain barrier to a higher degree than sumatriptan, in a subpopulation of MDD patients with melancholic depression (Whale et al. 2001).

Comparisons of 5-HT_1B_ receptor measurements in subjects with MDD with those of controls are fundamental for knowledge about this receptor in the pathophysiology of MDD. At present, information regarding 5-HT_1B_ receptor-related measures are available from studies of mRNA expression postmortem in forebrain regions (Anisman et al. 2008; Lopez-Figueroa et al. 2004) as well as PET studies of radioligand binding in vivo (Murrough et al. 2011b; Tiger et al. 2016).

PET offers molecular imaging of the living human brain at high anatomical resolution (Farde 1996). Brain PET requires a lipophilic ligand that can be labeled with a radioactive isotope and bind selectively to a target protein such as the 5-HT_1B_ receptor (Halldin et al. 2001). Tracer doses of the radioligand are injected intravenously, and a small portion passes the blood-brain barrier and bind to the target protein. The radioligand emits positrons that after a short distance form particle pairs with electrons. The particle pairs are annihilated and transformed into photons that traverse the skull and subsequently are detected by the surrounding PET system, enabling quantification of target protein binding in vivo in the brain. At present, there are two radioligands available for 5-HT_1B_ receptor imaging in humans: [^11^C]P943 and [^11^C]AZ10419369 (Zimmer and Le Bars 2013). Both [^11^C]AZ10419369 and [^11^C]P943 have high affinity for 5-HT_1B_ receptors (*K*_*D*_ = 0.4 and *K*_*D*_ = 1.2 nM, respectively) (Paterson et al. 2013).

Despite the methodological differences and the small number of cases and controls, previous postmortem and in vivo studies consistently support a trend of low levels of cerebral 5-HT_1B_ receptor related measures in unmedicated MDD subjects (Anisman et al. 2008; Murrough et al. 2011b; Tiger et al. 2016).

Interestingly, the pattern of low 5-HT_1B_ receptors appears to be most prominent in the limbic cortices, in regions of reported relevance for MDD, such as the anterior cingulate cortex and hippocampus (Mayberg 1997; Steele et al. 2007). Low 5-HT_1B_ receptor binding has been measured with PET in the anterior cingulate cortex (ACC), both in patients with recurrent MDD (Tiger et al. 2016) and in patients with MDD and posttraumatic stress syndrome comorbidity (Murrough et al. 2011a) (Table 1). Moreover, lower p11 mRNA and protein levels in MDD patients compared with controls have been found postmortem in ACC, a region with distinct coexpression of p11 and 5-HT_1B_ receptors (Egeland et al. 2011). However, in a recent [^3^H]AZ10419369 postmortem study allowing antidepressant medication MDD patients did not differ from controls in 5-HT_1B_ receptor binding in pregenual ACC (Veldman et al. 2017). The absence of difference in 5-HT_1B_ receptor binding in MDD in this autoradiography study could possibly be explained by pharmacological increase in 5-HT_1B_ receptor *BP*_ND_, as has been demonstrated in cortical regions after SSRI in healthy subjects (Nord et al. 2013), canceling out any low 5-HT_1B_ receptor binding in MDD patients. Furthermore, despite antidepressant treatment in 10 out of 12 patients, the MDD group stood out with low 5-HT_1B_ receptor binding in females compared with males, in contrast with the control group and patients with schizophrenia and bipolar disorder, respectively (Veldman et al. 2017).

**Table 1:** PET studies of 5-HT_1B_ receptor binding in MDD

Study	Medication	Sample size (*n*)	Radioligand	Method of quantitative analysis	Results
Murrough et al. 2011b	Antidepressant naive	MDD 10 HC 10	[^11^C]P943	Multilinear reference tissue model MRTM2	20% lower *BP*_ND_ in MDD vs HC in VS/VP (Cohen’s *d* 2.1)
Tiger et al. 2016	Unmedicated	MDD 10 HC 10	[^11^C]AZ10419369	Simplified Reference Tissue Model SRTM	No significant difference in VS or P in MDD vs HC. 20% lower *BP*_ND_ in ACC in MDD vs HC (Cohen’s *d* 1.3). 17% lower *BP*_ND_ in SGPFC in MDD vs HC (Cohen’s *d* 1.1). 32% lower *BP*_ND_ in hippocampus in MDD vs HC (Cohen’s d 1.2)
Murrough et al. 2011a	Unmedicated	PTSD 49 (PTSD only 34, PTSD+MDD 15) HC 27	[^11^C]P943	Multilinear reference tissue model MRTM2	9% lower *BP*_ND_ in ACC in PTSD vs HC (Cohen’s *d* 0.7). 11% lower *BP*_ND_ in amygdala in PTSD vs HC (Cohen’s *d* 0.6). 19% lower *BP*_ND_ in caudate in PTSD vs HC (Cohen’s *d* 0.8). 11% lower *BP*_ND_ in ACC in PTSD+MDD vs PTSD only^a^. 27% lower *BP*_ND_ in caudate in PTSD+MDD vs PTSD only^a^

*HC* healthy controls, *VS/VP* ventral striatum/ventral pallidum, *VS* ventral striatum, *P* pallidum, *ACC* anterior cingulate cortex, *SGPFC* subgenual prefrontal cortex, *PTSD* posttraumatic stress disorder

^a^*BP*_ND_ in PTSD+MDD vs PTSD only was not reported

Likewise, in the hippocampus, 5-HT_1B_ receptor binding was low in recurrent MDD as measured in vivo with PET (Tiger et al. 2016), and in patients with comorbidity of PTSD and MDD (Murrough et al. 2011a), and mRNA expression was numerically lower in parts of the hippocampus in MDD detected postmortem with in situ hybridization analysis (Lopez-Figueroa et al. 2004). Furthermore, in MDD subjects who had committed suicide, lower hippocampal 5-HT_1B_ receptor (in female subjects) and p11 mRNA expression compared with controls was found (Anisman et al. 2008). In the same study, low 5-HT_1B_ receptor and p11 mRNA expression was found in the frontopolar cortex. In the hypothalamus, by contrast, 5-HT_1B_ receptor mRNA expression was high in MDD suicide subjects (Anisman et al. 2008).

In the prefrontal cortex, 5-HT_1B_ receptor binding results so far have been negative, with no effect of MDD, neither in vivo with PET (Tiger et al. 2016) nor in membrane homogenates postmortem (Huang et al. 1999). Furthermore, in the basal ganglia, inconsistent findings have been reported in two PET studies. While Murrough et al. described low 5-HT_1B_ receptor binding in the ventral striatum/ventral pallidum in MDD (Murrough et al. 2011b), we could not replicate this difference between MDD patients and controls (Tiger et al. 2016). However, the MDD populations in the two studies differed in a number of aspects, with the first study including subjects that were smoking, subjects with higher BMI, and a larger proportion of subjects with no previous exposure to antidepressant drugs, in contrast with the subjects of the latter study. There were also technical differences in the two studies, such as the use of different 5-HT_1B_ receptor ligands, [^11^C]P943 (Murrough et al. 2011b) and [^11^C]AZ10419369 (Tiger et al. 2016), respectively, and different approaches in defining regions of interest (ROI), where Murrough et al. used a combined ROI from an automated template and we defined the ventral striatum and pallidum manually as separate ROIs. Perhaps most importantly, with the limited number of subjects in the two studies, there is a risk of type II errors, with failure to detect differences between MDD patients and controls due to low power.

In conclusion, low 5-HT_1B_ receptor binding and expression in ACC and hippocampus, key regions of the neurocircuitry of MDD (Mayberg 1997) has been reported.

### 5-HT_1B_ receptors and substance abuse

Loss of control is a core feature in substance abuse disorders. Loss of 5-HT_1B_ receptors in rodents induces a phenotype with reduced impulse control (Bouwknecht et al. 2001; Nautiyal et al. 2015). The involvement of 5-HT_1B_ receptors in abuse has mainly been studied in relation to alcohol and cocaine, though there are also reports relating to amphetamine effects (Miszkiel et al. 2011) and pathological gambling (Potenza et al. 2013).

Alcohol consumption has been reported to be high in 5-HT_1B_ receptor knockout mice (Crabbe et al. 1996). Correspondingly, low 5-HT_1B_ receptor densities have been demonstrated with autoradiography in cingulate and retrosplenial cortices, septum, and amygdala in alcohol-preferring rats (McBride et al. 1997). Furthermore, 5-HT_1B_ receptor agonists reduce alcohol self-administration in rats (Maurel et al. 1999; Tomkins and O'Neill 2000). On the other hand, overexpression of 5-HT_1B_ receptors in the nucleus accumbens in rats lead to increased alcohol consumption (Hoplight et al. 2006). In humans, associations between the 5-HT_1B_ receptor polymorphisms A161T in the 5′ regulatory region, with high reporter gene expression, and G681C and alcohol dependence (Sun et al. 2002) and antisocial alcoholism (Lappalainen et al. 1998), respectively, have been found. Additionally, high 5-HT_1B_ receptor binding in the ventral striatum in subjects with alcohol dependence has been demonstrated with PET (Hu et al. 2010). However, no differences between alcoholic and control subjects in 5-HT_1B_ receptor density in nucleus accumbens or any other examined brain region were found postmortem (Storvik et al. 2012). Furthermore, Huang et al. found no association between the 5-HT_1B_ receptor gene polymorphism G861C and alcoholism (Huang et al. 2003). Thus, despite neatly consistent small animal model data, the role of 5-HT_1B_ receptors in alcohol abuse remains unclear. This underscores the necessity of translational validation of animal model data in humans as a mandatory part of disease research.

Regarding 5-HT_1B_ receptors and cocaine, mice lacking the receptor self-administer cocaine to a higher degree (Castanon et al. 2000; Rocha et al. 1998). However, inhibition of 5-HT_1B_ receptors has either reduced or had no effect on cocaine self-administration (Miszkiel et al. 2011). When activating 5-HT_1B_ receptors, the acquired effect on the reinforcing effects of cocaine has been reported to depend on the current state in relation to cocaine intake. 5-HT_1B_ receptor agonists and 5-HT_1B_receptor gene transfer into the nucleus accumbens increase cocaine reinforcement during a self-administration period and decrease cocaine seeking and reinstatement of cocaine seeking behavior when given during abstinence (Neisewander et al. 2014; Pentkowski et al. 2012). Moreover, viral overexpression of 5-HT_1B_ receptors in nucleus accumbens reduces cocaine-seeking behavior elicited by cocaine priming (Nair et al. 2013). The 5-HT_1B_ receptor effect on cocaine reinforcement may relate to cocaine-induced receptor trafficking, as 5-HT_1B_ receptor mRNA increase in the dorsal striatum upon initiation of cocaine administration and decrease during abstinence (Neumaier et al. 2009). In humans, low 5-HT_1B_ receptor binding in vivo has indeed been described during abstinence not in striatum but in regions connected to the ventral striatum: anterior cingulate cortex, hypothalamus, and frontal cortex, in subjects with cocaine dependence (Matuskey et al. 2014).

### 5-HT_1B_ receptors and aggression

An early and distinct finding in 5-HT_1B_ receptor behavioral research has been its key role in regulating aggression, initially discovered through the enhanced aggressive behavior toward an intruder in 5-HT_1B_ receptor knockout mice (Bouwknecht et al. 2001; Olivier et al. 1995; Saudou et al. 1994). Indeed, stimulation of 5-HT_1B_ receptor signaling with the agonists CP 94,253, CP 93,129, and zolmitriptan administered intra-raphe or intra-peritoneally and anpirtoline injected intra-peritoneally has been reported to induce marked serenic effects in mice (de Almeida et al. 2001; Faccidomo et al. 2008; Faccidomo et al. 2012; Fish et al. 1999; Rilke et al. 2001). However, in mice with a history of alcohol self-administration, CP 94,253 increased the number of bite attacks when injected into the medial prefrontal cortex after the mice had drunk a high dose of alcohol (Faccidomo et al. 2008). The 5-HT_1B_ receptor-mediated aggression modulating effect may thus depend on the brain region, at least in alcohol-triggered aggressive behavior. Interestingly, Nautiyal et al. using a temporal receptor knockdown approach in mice demonstrated that forebrain 5-HT_1B_ heteroreceptor expression during the postnatal period regulate the development of aggressive traits, while neither adult receptor rescue nor whole life autoreceptor knockdown had any effect on aggressive behavior (Nautiyal et al. 2015). In humans, a polymorphism for the 5-HT_1B_ receptor genotype has been associated with childhood aggressive behavior and has been shown to influence the relationship between aggressive behavior during childhood and adult hostility (Hakulinen et al. 2013). Furthermore, in a recent PET study of violent offenders, psychopathy measurements and self-reported trait anger correlated with striatal 5-HT_1B_ receptor binding, although not in the control group (da Cunha-Bang et al. 2016).

## Effects of antidepressive treatments on 5-HT_1B_ receptors

Studies on 5-HT_1B_ receptors in relation to established treatments for depression provide a rich source of information on the potential role of the 5-HT_1B_ receptor as a target for antidepressant treatment. SSRIs is the group of antidepressants most studied in the 5-HT_1B_ receptor literature. In rats, time-dependent reduction of 5-HT_1B_ receptor mRNA has been reported at time points corresponding to onset of antidepressive effect in clinical studies (Gelenberg and Chesen 2000; Neumaier et al. 1996). In a follow-up study, the SSRI-induced reduction of dorsal raphe 5-HT_1B_ receptor mRNA expression was maintained after 8 weeks SSRI administration, but 5-HT_1B_ receptor mRNA levels were rapidly restored upon drug washout. Nortriptyline, however, had no effect on 5-HT_1B_ mRNA expression (Anthony et al. 2000). Similarly, chronic fluoxetine administration reduced 5-HT_1B_ receptor function in hippocampal and cortical neurons (Blier et al. 1988; Lifschytz et al. 2004) and induced downregulation of 5-HT_1B_ receptors in suprachiasmic nucleus in rats (O'Connor and Kruk 1994). Furthermore, in 5-HT_1B_ receptor gene knockout mice, the SSRI effect on forced swimming test immobility was absent, suggesting 5-HT_1B_ receptors as necessary for mediating the antidepressant effect of SSRI (Trillat et al. 1998). However, reduced depression-like behavior has been demonstrated with fluoxetine in 5-HT_1B_ receptor knockout mice using another model of depression, the tail suspension test (Mayorga et al. 2001). Moreover, pharmacological 5-HT_1B_ receptor inhibition reduced the mobility-enhancing effect of SSRI, but not of imipramine, in the forced swim test (Chenu et al. 2008). However, reduced anti-immobility time in the forced swim test with SSRI in combination with 5-HT_1B_ receptor antagonists has also been reported (Tatarczynska et al. 2002). Moreover, in a similar study by the same group, no effect of combined administration of SSRI and 5-HT_1B_ receptor antagonists on the forced swim test was found, although rats given 5-HT_1B_ receptor antagonists together with tricyclic antidepressants or a selective monoamine oxidase inhibitor displayed less immobility in the forced swimming test (Tatarczynska et al. 2004a). On the other hand, activation of 5-HT_1B_ receptors with the agonists anpirtoline and RU 24969 potentiated the antidepressive-like effect of SSRIs as well as imipramine and noradrenaline reuptake inhibitors in the forced swim test in mice (David et al. 2001; Redrobe et al. 1996), albeit in the case of anpirtoline only in young, but not in old, mice. In humans, a marked reduction of zolmitriptan-induced rise in GH concentration has been reported in MDD patients after SSRI treatment, possibly due to downregulation of 5-HT_1B/1D_ receptors after SSRI (Whale et al. 2001). Furthermore, a polymorphism for the 5-HT_1B_ receptor gene was associated with SSRI response in samples of depressed patients from the STAR*D study (Villafuerte et al. 2009). Moreover, early reduction of the functionally related protein p11 in natural killer cells and monocytes with SSRI treatment has been found to correlate with antidepressant response in patients with MDD (Svenningsson et al. 2014). Altogether, in most studies, reduction of 5-HT_1B_ receptor expression and function has been reported with SSRI, while paradoxically 5-HT_1B_ receptor agonists consistently display antidepressant-like effects in animal models. One explanation could be that 5-HT_1B_ receptors are internalized upon activation, leading to downregulation of 5-HT_1B_ receptors, especially in the raphe nuclei neurons, which have relative few synapses and therefore are more sensitive to fluctuations in synaptic serotonin concentrations (Jacobs and Azmitia 1992).

Although the main body of research on antidepressants’ effect on 5-HT_1B_ receptors consists of studies with SSRI, a number of other drugs used in the treatment of depression have been examined in relation to 5-HT_1B_ receptors such as the tricyclic antidepressants and the monoamine oxidase inhibitor mentioned earlier in the text. Lithium, one of the most established drugs for the treatment of mood disorders, has been suggested to act through 5-HT_1B_ receptors, based on its potentiating effects on 5-HT_1B_ receptor agonists in the forced swimming test (Redrobe and Bourin 1999). Interestingly, in a study in rodent cortices and blood platelets from healthy human subjects, lithium in “therapeutic” concentrations relatively selectively and noncompetitively inhibited 5-HT_1B_ receptor binding and actions, such as G-protein coupling and reduction of forskolin-induced cAMP release (Massot et al. 1999). Following up these results in a small group of depressed patients, Januel et al. described a similar blocking effect on 5-HT_1B_ receptor-induced cAMP reduction with lithium treatment. Furthermore, the reduced adenylate cyclase activity inversely correlated with antidepressive treatment response (Januel et al. 2002), indicating 5-HT_1B_ receptor involvement in lithium treatment effect. Lithium inhibits glycogen synthase kinase-3 (GSK3) activity (Stambolic et al. 1996). In a previous study, GSK3 inhibitors and molecular ablation of GSK3β abolished 5-HT_1B_ receptor coupling to G_i_α_2_ subunits and GSK3 inhibitors abolished 5-HT_1B_ receptor-mediated inhibition of serotonin release in the mouse cortex (Chen et al. 2011). Thus, the regulation of 5-HT_1B_ receptor function by lithium could possibly be mediated via GSK3 inhibition. However, 5-HT_1B_ receptor involvement is not limited to the established antidepressants. In the most promising new line of MDD drug research, a glutamate α-amino-3-hydroxy-5-methylisoxazole-4-propionic acid (AMPA) receptor-dependent increase in 5-HT_1B_ receptor binding in the nucleus accumbens and ventral pallidum has been demonstrated in response to ketamine in nonhuman primates (Yamanaka et al. 2014).

In addition, a number of 5-HT_1B_ receptor studies of other treatment modalities have been performed. Different brain stimulation regimes have been examined in rats. Reduced serotonin increase in response to the 5-HT_1B_ receptor antagonist GR 127935 has been demonstrated with repetitive transcranial magnetic stimulation (Gur et al. 2000). Electroconvulsive shock (ECS), the animal model of electroconvulsive therapy (ECT), on the other hand, did not alter 5-HT_1B_ autoreceptor-mediated action in the hypothalamus or hippocampus (Gur et al. 2002). However, a significant increase of the 5-HT_1B_ receptor-related p11 has been demonstrated in the cortex after ECS (Svenningsson et al. 2006). In humans, the literature on brain 5-HT_1B_ receptor response to treatment for depression is very limited. In the only 5-HT_1B_ receptor PET study of MDD treatment published so far, a distinct reduction of 5-HT_1B_ receptor binding in the dorsal brain stem, encompassing especially the median raphe nucleus, was observed after successful cognitive behavioral therapy (Tiger et al. 2014).

## Targeting the 5-HT_1B_ receptor for antidepressant effect

The 5-HT_1B_ receptor can be relatively labile in its expression, with rapid and region-specific changes in mRNA and protein expression in response to conditions such as stress (Neumaier et al. 1997), drug exposure (Anthony et al. 2000; Neumaier et al. 1996; Nord et al. 2013), and estrogen status (Hiroi and Neumaier 2009). This lability is probably an important factor in the diversity of behavioral responses that have been reported for pharmacological studies of this receptor. Sensitivity to stress and pharmacological manipulations makes the 5-HT_1B_ receptor particularly interesting as a target for treatment of MDD, serving as a marker for state rather than trait. A multitude of drugs targeting the 5-HT_1B_ receptor have demonstrated potential antidepressant properties. Which is then the most suitable approach to modulate 5-HT_1B_ receptor activity in order to best treat depression?

Given the inhibitory effect of 5-HT_1B_ receptors on serotonin release and the predominantly prevailing serotonin hypothesis of MDD, it makes sense to block 5-HT_1B_ receptors for antidepressant effect (Slassi 2002). Thus far, most 5-HT_1B_ receptor drug candidates for MDD treatment have been antagonists, such as SB-616234-A (Dawson et al. 2006), AZD3783 (Zhang et al. 2011), and AR-A000002 (Hudzik et al. 2003). It has been proposed that 5-HT_1_ receptor activation counteracts the serotonin-enhancing effects of SSRI and thereby contribute to the latency of therapeutic effect (Blier and de Montigny 1994; Nutt 2002). SSRI-induced downregulation of 5-HT_1_ receptors would then restore the serotonin-elevating effects of the drugs and hence enable a clinical effect, providing a rationale for blocking 5-HT_1B_ receptors for rapid antidepressant response. However, the serotonin-releasing and reuptake inhibiting drug fenfluramine had no augmenting effect compared to placebo in patients with treatment refractory depression (Price et al. 1990), even though it is a far more potent serotonin enhancer than any SSRI (Rothman and Baumann 2002). Moreover, 5-HT_1B_ receptor antagonists alone are likely not sufficiently effective as antidepressants, but likely have a potential as adjuvants in the treatment for depression (Ruf and Bhagwagar 2009). Hence, a combination of SSRI and 5-HT_1B_ receptor antagonist has been introduced as a new antidepressant concept (Matzen et al. 2000).

If, on the other hand downregulation/desensitization of 5-HT_1B_ autoreceptors rather is mediating the antidepressant effects of SSRI, agonists would constitute a rational treatment approach, promoting quicker and more selective downregulation of 5-HT_1B_ receptors. Moreover, treating depression by stimulating forebrain 5-HT_1B_ receptors would be in line with the low 5-HT_1B_ receptor binding found in patients with MDD, especially in the limbic system, in areas of reported importance in the pathophysiology of depression (Mayberg 1997; Steele et al. 2007). Indeed, increased 5-HT_1B_ receptor binding in serotonergic projection areas has been demonstrated in humans after a single, clinically relevant, dose of SSRI (Nord et al. 2013). Furthermore, there was a trend of increased 5-HT_1B_ receptor mRNA in projection areas after chronic SSRI administration in rats (Neumaier et al. 1996). Interestingly, in both humans and rats, 5-HT_1B_ receptor measurements were reduced in raphe nuclei with SSRI (Neumaier et al. 1996; Nord et al. 2013). The increase in serotonin concentration with SSRI could thus primarily downregulate raphe nuclei 5-HT_1B_ receptors due to the relatively low number of serotonergic synapses and therefore higher serotonin concentration in each synapse in this region (Jacobs and Azmitia 1992). This would, in theory, lead to increased serotonin release in projection areas and possibly upregulation of inhibitory 5-HT_1B_ receptors in these regions, potentially counteracting the low 5-HT_1B_ receptor levels earlier described in patients with MDD (Anisman et al. 2008; Murrough et al. 2011a; Murrough et al. 2011b; Tiger et al. 2016). Moreover, 5-HT_1B_ receptor agonists have been proven successful in animal models for depression (see “Effects of agonists and antagonists in animal models of depression” section). However, treatment with 5-HT_1B_ receptor agonists entail a potential risk of cognitive side effects, given the impaired spatial reference memory after 5-HT_1B_ receptor agonist administration in rats and the superior spatial reference memory performance in 5-HT_1B_ receptor gene knockout mice (Buhot et al. 2000; Woehrle et al. 2013).

In this sense, partial agonists offer an appealing alternative. Indeed, this concept has been successfully demonstrated in the treatment of depression with the partial 5-HT_1B_ receptor agonist vortioxetine (Berhan and Barker 2014). However, vortioxetine also inhibits serotonin reuptake and exerts multiple serotonergic actions at additional receptor subtypes (Dhir and Sarvaiya 2014; Sanchez et al. 2015). Thus, it remains unknown whether the partial agonist effect at the 5-HT_1B_ receptor represents a primary mechanism necessary for the antidepressant properties.

Finally, inverse agonism should also be an interesting approach, although to our knowledge currently no studies of the antidepressant effects of selective 5-HT_1B_ receptor inverse agonists are available. In contrast with antagonists, 5-HT_1B_ receptor inverse agonists can increase serotonin release per se, as demonstrated with the 5-HT_1B_ receptor inverse agonist SB-236057-A in the hippocampus in guinea pigs (Roberts et al. 2001). The potential of this mode of action is underscored by the 5-HT_1B_ receptor inverse agonist properties of lithium, outlined in “Effects of antidepressive treatments on 5-HT1B receptors” section. A drug which mimics the effect of lithium, hopefully without the nephrotoxic effects and narrow therapeutic interval of the latter, would be a major contribution to the treatment of mood disorders (Curran and Ravindran 2014; Nelson et al. 2014; Raja 2011).

## Conclusion

The 5-HT_1B_ receptor is a key protein in mice and men, modulating a number of physiological functions and behaviors through regulation of release of serotonin and a number of other neurotransmitters. The main body of the literature on 5-HT_1B_ receptors in relation to depression consists of research in animals, although data from human studies on 5-HT_1B_ receptor in MDD are slowly accumulating. The evidence summarized above supports a role of the 5-HT_1B_ receptor as an interesting target for antidepressant treatment. However, it is not yet clear how to best alter 5-HT_1B_ receptor binding and action, for optimal effect, although it could be argued that 5-HT_1B_ receptor agonists have an advantage given the low 5-HT_1B_ receptor binding described in MDD patients and the increase in 5-HT_1B_ receptor binding with SSRI in humans, in serotonergic projection areas. So far, proof of concept for 5-HT_1B_ receptor-mediated MDD treatment effect has been demonstrated for the partial 5-HT_1B_ receptor agonist vortioxetine, although effects at additional 5-HT-binding sites likely contribute to the antidepressant effects of this compound. Future studies on the effect of established treatments for depression, such as antidepressants and ECT, on 5-HT_1B_ receptor binding and action are needed to guide the process of developing drugs targeting the 5-HT_1B_ receptor, with potential antidepressant effect. In the process of 5-HT_1B_ receptor drug development, it is important to be aware of the well-characterized species differences in receptor pharmacology.
